# Audiology in 2050: Aligning Collective Professional Perspectives to Create a Roadmap for the Future

**DOI:** 10.1055/s-0046-1825796

**Published:** 2026-10-06

**Authors:** Amyn M. Amlani, Victor Bray

**Affiliations:** 1 Department of Communication Disorders, College of Allied Health, University of Oklahoma Health Sciences Center, Oklahoma City, Oklahoma, United States; 2 Osborne Audiology Department, College of Nursing and Health Professions, Drexel University, Elkins Park, Pennsylvania, United States

**Keywords:** professional autonomy, Medicare LLP status, scope of practice, health policy, workforce sustainability

## Abstract

This study examines expert perspectives on the future of the audiology profession using the Audiology 2050 framework as a guiding analytic lens. A structured, open-ended survey was distributed to 52 invited subject matter experts representing eight professional segments of the audiology workforce, with 41 responding (79%). Responses were analyzed using two-stage qualitative analysis: inductive thematic coding followed by framework-based recoding across the seven Audiology 2050 domains. Findings reveal that the profession's most frequently cited challenges — scope limitations, inconsistent practice standards, workforce strain, reimbursement barriers, and constrained autonomy — are not independent problems but structurally interdependent system components that must mature in a coordinated sequence. Scope of practice modernization emerges as the foundational enabling condition upon which evidence-based practice standardization, workforce redesign, professional socialization, and advocacy collectively depend. Medicare Limited License Practitioner (LLP) reclassification is identified as the critical structural inflection point, functioning as a multiplier of upstream alignment rather than a standalone solution. Primary care entry point status for auditory and vestibular conditions is positioned as the culminating, profession-wide outcome. These findings are synthesized into a proposed phased roadmap to 2050 and a dependency network illustrating the sequencing required for coordinated structural reform. The analyses suggest that professional autonomy is achievable, but only if dependency relationships among foundational domains are acknowledged and addressed in sequence.

## Introduction

In recent decades, audiology has experienced substantial advancements in educational standards, clinical practice, and technological innovation. The adoption of the Doctor of Audiology (AuD) degree, rapid advances in diagnostic and rehabilitative tools, and a growing body of evidence linking hearing and balance to broader health outcomes have positioned audiology as an increasingly relevant component of contemporary healthcare. Yet despite these advances, the profession continues to experience persistent structural challenges related to workforce sustainability, economic viability, scope of practice, and professional identity. Collectively, these pressures suggest that audiology is not simply confronting isolated operational concerns but rather navigating a critical inflection point that will shape its future role within the healthcare system.

This issue of *Seminars in Hearing* examines the audiology profession through a set of interrelated lenses, including workforce supply and utilization, earnings and compensation, educational debt burden, professional taxonomy and scope, and the future design of AuD education. Rather than treating these domains as independent variables, the contributions collectively frame them as components of a single professional ecosystem. From this perspective, workforce challenges are not reducible to educational models alone, nor are economic pressures separable from regulatory constraints or curricular structure. Instead, the manuscripts converge on a central theme: audiology's long-term sustainability depends on structural alignment between professional preparation and the regulatory, economic, and reimbursement conditions that govern its application.

Across these domains, a consistent pattern emerges—one documented in detail across the companion analyses in this issue. In the United States, audiology has successfully transitioned to doctoral-level education without achieving full doctoral-level integration into healthcare delivery systems. Audiologists are trained to manage complex hearing and balance conditions across the lifespan, yet often remain constrained by limited clinical authority, inconsistent scope of practice, and reimbursement structures that undervalue professional services provided. These misalignments have downstream consequences for workforce participation, career longevity, and access to care. Importantly, they also shape how audiology is perceived by policymakers, healthcare administrators, and the public—as technical support rather than a fully realized autonomous clinical profession.

It is within this context that Audiology 2050 functions as a set of seven aspirational goals, in independent domains, for the profession to achieve over the next 25 years. First articulated by the Academy of Doctors of Audiology (ADA), Audiology 2050 has been used in this analysis as a profession-wide analytic lens rather than as an organization-specific initiative. 1 Its core principles—beginning with professional autonomy and including workforce sustainability, full scope of practice, equitable reimbursement, and integration into public health—reflect challenges and aspirations consistently voiced by audiologists, independent of organizational affiliation. As such, Audiology 2050 should be understood not as one organization's initiative, but as shared infrastructure for the structural alignment required to support audiology's evolution into an autonomous doctoring profession.

Fifty-two subject matter experts were invited to provide perspectives on the future of the audiology profession using the Audiology 2050 framework as a guided reference to facilitate discussion. Invitees were purposively selected to reflect a broad range of professional experience and organizational membership, including current AuD students; early-, mid-, and late-career audiologists; and representation across private practice, Veterans Administration (VA), hospital-based, otolaryngology, hearing aid industry, educational, and academic settings, with invitees drawn from across the major audiology professional organizations rather than from any single organizational constituency. The present manuscript draws on qualitative input from 41 respondents ( Fig. 1 ). Through thematic analysis and subsequent framework-based recoding, these perspectives were examined as indicators of alignment or misalignment between the profession's past and present conditions and its stated strategic objectives for the future. This approach allows collective professional sentiment to be interpreted beyond aspirational goals as empirical insight into the structural conditions shaping audiology's trajectory.

**Fig. 1:**
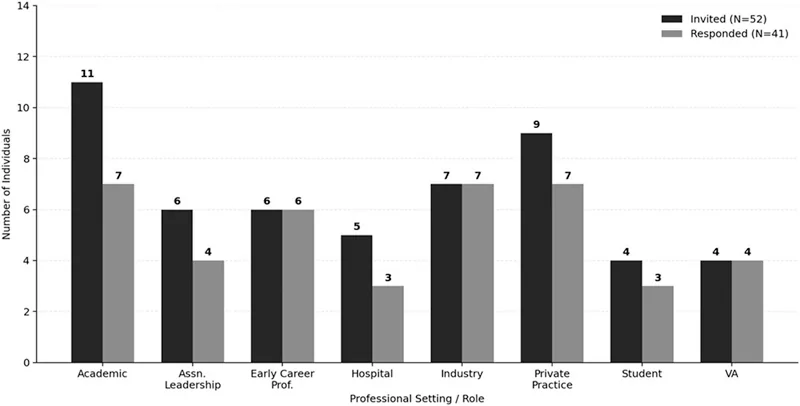
Distribution of invited and responding subject matter experts by professional group.

Within the context of this issue, this manuscript serves an integrative function. The central contribution of this analysis is not the identification of new challenges, but the clarification that existing challenges are structurally interdependent and must be addressed in coordinated actions rather than as isolated initiatives. It synthesizes workforce-related challenges described across multiple occupational domains and situates them within a shared strategic framework, clarifying how education, workforce design, regulatory modernization, and federal policy function as interdependent system components whose alignment must occur in a coordinated sequence. This analysis is intentionally bound by its qualitative design: the thematic findings confirm and contextualize structural patterns documented through quantitative methods in companion analyses—they do not independently establish causal relationships, generate prevalence estimates, or produce generalizable findings beyond the purposive expert sample. The structural claims advanced in the Discussion are grounded in the convergence of qualitative perspectives with the quantitative evidence developed elsewhere in this issue; neither source alone is treated as sufficient. From this perspective, achieving the goals articulated in the Audiology 2050 framework is necessary to prevent further erosion of workforce capacity, professional identity, and audiology's contribution to public health.

Overall, this special issue suggests that audiology's future will depend less on technological advancement than on strategic coherence. Aligning collective professional perspectives around a shared roadmap offers an opportunity to move beyond fragmented solutions toward a unified, autonomous future. By examining where the profession currently stands in relation to the Audiology 2050 framework, this manuscript seeks to clarify not only the direction of change but also the structural commitments required to achieve it. To examine this alignment systematically, the following sections describe the qualitative methods used to elicit and analyze expert perspectives and the framework-based approach by which these insights were mapped to the Audiology 2050 framework.

## Methods

### Study Design and Purpose

This study employed a qualitative, framework-based analytic design to examine how expert perspectives on the audiology workforce align with the profession's long-term strategic objectives. The primary aim was not to generate new theory, but to synthesize informed professional judgment and assess structural alignment between lived professional experience and the Audiology 2050 framework. Qualitative methods were selected to capture nuance, complexity, and contextual insight that are not readily accessible through quantitative workforce metrics alone.

### Participants and Sampling

Participants consisted of 41 subject matter experts with demonstrated leadership and expertise across multiple sectors of the audiology profession. Experts were purposively sampled to ensure representation across clinical practice settings (private practice, hospital-based, and academic clinics), academia, professional advocacy, workforce analysis, and policy engagement. This approach was intended to capture a broad range of perspectives reflective of the profession's structural diversity rather than to achieve statistical generalizability.

Participants were invited based on their professional roles, contributions to profession-related initiatives, and familiarity with systemic challenges facing audiology. All participants provided responses voluntarily, and no identifying information was retained for analysis.

### Data Collection

Data were collected through a structured, open-ended survey distributed electronically between September 1, 2025, and October 31, 2025. Survey questions were designed to elicit expert perspectives on current and future workforce challenges, professional sustainability, scope of practice, and strategic priorities for the profession. Audiology 2050 was provided as a guiding analytic framework to anchor responses around shared strategic domains, enabling systematic cross-respondent comparison without constraining individual perspectives.

Open-ended questions encouraged respondents to reflect broadly on structural barriers, opportunities for reform, and necessary conditions for achieving a unified and autonomous future for audiology. This design allowed participants to emphasize issues they viewed as most consequential, rather than constraining responses to predefined categories.

### Analytic Approach

Qualitative analysis proceeded in two stages. First, an inductive thematic analysis was conducted to identify recurrent concepts and patterns across responses. Initial coding focused on capturing salient ideas expressed by participants, with attention to issues related to professional autonomy, workforce sustainability, scope of practice, reimbursement, education, and professional identity. Codes were iteratively refined through repeated review to ensure internal consistency and conceptual clarity.

In the second stage, identified themes were recoded using Audiology 2050 as an analytic framework. This framework-based recoding enabled examination of how emergent professional concerns aligned—or failed to align—with the roadmap's core domains, including scope of practice consistent with clinical doctoring, standardized evidence-based care, workforce design and utilization, professional socialization and advocacy, economic viability, federal recognition, and primary care entry point roles. This two-step method preserved inductive insights while situating them within a unified strategic framework.

To support systematic organization of qualitative responses, an artificial intelligence (AI)–assisted large language model (ChatGPT, OpenAI) was used solely to assist in organizing qualitative responses; all thematic interpretation and conclusions were determined by the authors.

### Rigor and Trustworthiness

Multiple strategies were implemented to enhance analytical rigor and trustworthiness. Purposive expert sampling strengthened credibility by ensuring informed, experience-based perspectives. Framework-based recoding improved dependability by providing a transparent and replicable structure for interpretation. Throughout the analysis, emphasis was placed on identifying convergent themes rather than isolated or idiosyncratic comments, with priority given to patterns that emerged consistently across respondents. Given the exploratory and synthesis-focused nature of this work, findings are intended to inform strategic alignment and policy discourse rather than estimate prevalence or establish causal relationships.

### Ethical Considerations

This study involved analysis of anonymous professional opinion data and did not include patient information or clinical intervention. As such, it was considered exempt from institutional review board oversight. Participation was voluntary, and responses were analyzed in aggregate to protect confidentiality.

## Results

Qualitative results are presented according to the seven domains of the Audiology 2050 framework and are organized to mirror the framework used for analysis.

**1. Scope of practice consistent with other clinical doctors**

Respondents consistently identified scope of practice as a foundational issue shaping audiology's ability to function as a clinical doctorate. Across responses, participants described a persistent misalignment between doctoral-level education and the regulatory, reimbursement, and institutional constraints that govern audiology practice. This misalignment was framed not as a lack of competence, but as the result of externally imposed limitations that restrict how audiologists are permitted to apply their training in clinical settings.

### Training–Authority Mismatch

A dominant theme centered on the disconnect between the breadth of audiology training and the authority granted to practice independently. Respondents emphasized that audiologists are educated to diagnose, manage, and monitor hearing and balance disorders, yet are often constrained by scope language that limits ordering of diagnostic tests, prescribing authority, and independent clinical decision-making. Multiple participants observed that these constraints remain in effect even though audiologists demonstrate the necessary competencies, characterizing limitations on scope as “artificial” or “arbitrary” rather than clinically justified.

### Setting-Dependent Scope and Proof-of-Concept Environments

Scope of practice was repeatedly described as highly variable across practice environments. Respondents cited VA settings as proof-of-concept models in which audiologists function with expanded clinical authority, including independent entry points for auditory complaints, the ability to order laboratory tests, limited prescribing authority, and unrestricted cerumen management. In contrast, audiologists practicing in hospital-based, otolaryngology-owned, or Medicare-dependent environments frequently reported a constrained scope tied to physician oversight, billing structures, or institutional policy. This variability was perceived as evidence that scope limitations are policy-driven rather than training-driven.

Critically, respondents did not frame the VA model as an aspirational ideal, but as an operational reality already functioning at scale within a major healthcare system. In this sense, the policy barriers to full-scope practice are not hypothetical—they are the central obstacle separating current audiology practice from a model that already demonstrably works at scale.

### Professional Identity and the “Nonmedical” Designation

Many respondents raised concerns that statutory and regulatory language designating audiology as “nonmedical” may compromise the profession's identity and clinical legitimacy. Participants argued that hearing and balance evaluations inherently involve medical necessity, citing comprehensive case history, differential diagnosis, and management of conditions associated with illness, injury, or functional impairment. Continued use of “routine” or “nonmedical” descriptors was viewed as self-limiting, contributing to reimbursement barriers and reinforcing external perceptions of audiology as technical support rather than a clinical discipline.

### Cautious Support for Scope Expansion Without Overreach

Respondents favored aligning audiology scope of practice with that of other clinical doctoring professions, while emphasizing the importance of precision and restraint. Some cautioned against characterizing audiologists as primary care providers for overall health, instead stressing that professional independence should remain focused on the management of auditory and vestibular disorders. These respondents underscored that expanded scope should reflect demonstrated competence and licensure boundaries, distinguishing appropriate clinical authority from encroachment into domains traditionally managed by physicians, nurse practitioners, or physician assistants.

### Scope as a Precondition for Downstream Reform

Across responses, scope of practice was implicitly positioned as a prerequisite for other strategic objectives, including entry point status, Medicare Limited License Practitioner (LLP) reclassification, and reimbursement reform. Respondents suggested that without scope alignment consistent with clinical doctorate training, efforts to improve workforce utilization, economic sustainability, or public recognition would remain constrained. In this framing, scope of practice was not viewed as an endpoint, but as enabling infrastructure necessary for the realization of broader professional goals.

### Alignment Statement

Collectively, these findings indicate substantial misalignment between doctoral-level audiology training and the scope of practice permitted across many clinical environments. This misalignment underscores the necessity of modernizing the scope of practice as a foundational condition for advancing professional autonomy within the Audiology 2050 framework. Respondents consistently emphasized that:

- Standardization of training, including continued universal incorporation of areas such as biomedicine, pharmacology, and imaging, would strengthen professional readiness and support responsible scope modernization.
- Although audiologists are trained to evaluate, diagnose, manage, and treat auditory and vestibular conditions, state scope language and institutional policies often recognize only portions of this continuum.
- Removal of “nonmedical” terminology from statutory and regulatory language was viewed as a necessary step toward aligning scope definitions with doctoral-level training.
- Scope alignment is necessary for audiologists to practice across the full continuum of care, including prevention, early identification, intervention, and long-term management.

**2. Standardized, evidence-based practice**

Respondents consistently emphasized standardized, evidence-based practice as a core requirement for professional credibility, autonomy, and public trust. Across responses, participants expressed strong support for evidence-based practice as a defining attribute of a clinical doctorate, while simultaneously identifying substantial gaps between existing evidence, clinical training, and routine implementation. These gaps were framed as both cultural and structural challenges for the profession.

### Evidence Generation Without Consistent Translation

A recurrent theme involved concern that audiology has produced a robust and growing evidence base, yet has struggled to translate that evidence into consistent clinical practice. Respondents noted that while audiologists possess the knowledge and skills to differentially diagnose and manage auditory and vestibular disorders, these competencies are not uniformly applied across practice settings. Several participants expressed frustration that established evidence related to tinnitus assessment, vestibular evaluation, cochlear–retrocochlear differentiation, and rehabilitative counseling is frequently underutilized in routine care.

### Variability in Practice Standards Across Settings

Participants highlighted wide variability in adherence to evidence-based practices depending on employment context, payer mix, and business model. Audiologists practicing in environments driven by product sales or productivity metrics were perceived as less able—or less incentivized—to implement comprehensive, guideline-based care. In contrast, settings with clearer separation between clinical services and product transactions were viewed as more conducive to evidence-based decision-making. This variability was described as undermining both patient outcomes and the profession's claim to clinical authority.

### Standardization as a Shared Professional Responsibility

Numerous respondents supported the need for standardized practice expectations while cautioning against equating evidence-based standards with any single credentialing or accreditation pathway. Participants expressed concern that treating one approach as the default standard could alienate segments of the profession and impede broader adoption of best practices. Instead, standardization was viewed as a profession-driven responsibility—one that should be transparent, inclusive, and grounded in universally accepted clinical evidence rather than affiliation with any particular pathway.

### Economic and Reimbursement Barriers to Evidence-Based Care

Multiple respondents linked variability in evidence-based practice to reimbursement models that inadequately recognize or incentivize comprehensive clinical assessment and management. Participants noted that when payers prioritize procedural throughput or product delivery over diagnostic reasoning and longitudinal care, audiologists are structurally disincentivized from implementing best practices. In this context, departures from evidence-based care were framed not as professional negligence, but as adaptive responses to misaligned economic incentives.

### Standardization as a Prerequisite for Autonomy and Trust

Across responses, standardized, evidence-based practice was positioned as foundational to achieving professional autonomy. Respondents emphasized that claims for expanded scope, independent practice authority, and Medicare LLP reclassification must be accompanied by demonstrable consistency in care delivery. Without reliable adherence to evidence-based standards, participants expressed concern that efforts toward autonomy could be undermined by variability in practice quality and skepticism from payers and policymakers.

### Alignment Statement

Collectively, these findings indicate broad professional support for standardized, evidence-based practice as a defining attribute of a clinical doctorate, while also revealing persistent structural and cultural barriers to consistent implementation across practice settings. Respondents emphasized that:

- Although a robust evidence base exists in audiology, the absence of uniformly adopted, up-to-date standards of care and clinical guidelines contributes to wide variability in practice quality and clinical decision-making.
- Inconsistent translation of evidence into routine care is driven in part by reimbursement models, productivity pressures, and business structures that deprioritize comprehensive evaluation and longitudinal management.
- Standardization is a profession-wide responsibility rather than an organizational mandate, with concern that conflating evidence-based practice with endorsement of a single entity could impede broad adoption.
- Mechanisms that normalize and reinforce evidence-based care—such as accreditation of clinical practices—are valuable when positioned as transparent, inclusive, and grounded in widely accepted clinical standards.

The concern about organizational branding suggests that standards and accreditation are best positioned as profession-owned infrastructure rather than association-specific initiatives. Taken together, these perspectives suggest that advancement toward standardized, evidence-based practice within the Audiology 2050 framework will require not only continued guideline development, but also structural supports that incentivize and normalize their adoption across diverse practice environments.

**3. Professional workforce, including extenders**

Respondents consistently identified workforce configuration as a central determinant of audiology's sustainability and clinical reach. Across responses, participants emphasized that current workforce challenges are driven less by absolute shortages of audiologists than by inefficient utilization of doctoral-level expertise, uneven practice models, and the absence of a formally structured extender workforce. Within this context, the inclusion of audiology assistants and other service extenders was widely framed as necessary to support access, quality, and professional longevity.

### Underutilization of Doctoral-Level Expertise

A dominant theme involved concern that audiologists are frequently required to perform tasks that do not require doctoral-level training, limiting their capacity for complex diagnostic evaluation, counseling, and interdisciplinary care. Respondents described this underutilization as contributing to professional dissatisfaction and burnout, particularly among mid-career clinicians. Multiple participants observed that, in the absence of redistribution of routine clinical and operational responsibilities, expanding scope and evidence-based care expectations could further exacerbate pressures on an already constrained workforce.

### Support for Audiology Assistants and Tiered Care Models

There was strong and consistent support for the development of audiology assistants and other service extenders operating under an audiologist's supervision. Respondents emphasized that extender roles should be intentionally designed, competency-based, and led by audiologists to ensure alignment with professional values and standards of care. Participants highlighted the potential for tiered service delivery models to improve access in underserved settings, expand capacity in communal living environments, and allow audiologists to focus on services requiring advanced clinical judgment.

### Training, Credentialing, and Oversight Requirements

Many respondents cautioned that workforce expansion through extenders must be accompanied by standardized training, credentialing, and audiologist-led oversight, consistent with supervisory models used by other doctoring professions for advanced practice extenders. Participants expressed concern that inconsistent preparation or the absence of national competency frameworks could compromise care quality and undermine professional credibility. Several respondents advocated for formal certification pathways, national competency models, and accreditation of clinical training sites, drawing parallels to structured workforce models used in other health professions in which the supervising clinician retains responsibility for scope, delegation, and clinical decision-making.

### Attrition, Burnout, and Workforce Retention

Workforce sustainability was frequently discussed in relation to attrition and burnout. Respondents perceived that a meaningful proportion of audiology graduates exit the profession within the first decade of practice, attributing this trend to limited advancement opportunities, financial pressures, and role compression. In this framing, service extenders were viewed not as replacements for audiologists, but as a mechanism to preserve the workforce by enabling clinicians to practice at the top of their license and training.

### Attrition as a Compounding Workforce Crisis

Beyond burnout and dissatisfaction, several respondents flagged attrition—the outright departure of licensed audiologists from the profession—as a distinct and underexamined dimension of workforce sustainability. Estimates raised by participants suggested that a substantial proportion of AuD graduates exit clinical practice within the first decade following degree completion, representing a significant loss of training investment and workforce capacity that compounds the utilization challenges already described. Respondents characterized attrition as both a cause and a consequence of structural misalignment elsewhere in the profession: when scope is constrained, reimbursement is inadequate, and professional identity remains ambiguous, clinicians with the credentials to practice in adjacent sectors have little reason to remain in clinical audiology. Addressing attrition, in this framing, is not separable from the broader alignment agenda—it is one of its most measurable downstream consequences and a direct indicator of whether structural reform is producing results in the workforce.

### Proof-of-Concept Models and System-Level Opportunities

Several participants cited VA environments and large healthcare systems as examples where structured workforce models—including the integration of assistants and expanded audiologist authority—have improved efficiency and access to services. These environments were cited as evidence that tiered care models can be implemented without compromising quality or professional identity. Respondents emphasized that broader adoption would require alignment across state regulations, payer recognition, and educational preparation.

### Alignment Statement

Collectively, these findings indicate strong professional support for workforce models that enable audiologists to practice at the top of their license and training while expanding access through the appropriate use of service extenders. Respondents emphasized that:

- Current workforce challenges are driven less by a shortage of audiologists than by underutilization of doctoral-level expertise and inefficient care models.
- Redistribution of routine clinical and operational tasks to trained extenders is essential to preserving audiologists' capacity for complex diagnostic evaluation, management, and interdisciplinary care.
- Support for audiology assistants and other extenders is contingent on standardized training, clearly defined scope, and audiologist-led supervision to ensure quality and professional accountability.
- Variability in state regulations, credentialing pathways, and payer recognition remains a barrier to scalable workforce redesign.
- Structured workforce models implemented in settings such as the VA serve as proof-of-concept environments demonstrating that tiered care can improve access and efficiency without compromising professional identity.

Together, these perspectives suggest that workforce redesign within the Audiology 2050 framework depends on aligning scope, training, and regulatory infrastructure to support extender models that enhance—rather than dilute—doctoral-level audiology practice.

**4. Audiologists advance professional socialization initiatives**

Respondents consistently emphasized professional socialization as a critical yet underdeveloped component of audiology's evolution as a clinical doctorate. Across responses, participants described gaps in how audiologists are prepared to assume professional identity, responsibility, and leadership commensurate with doctoral-level training. These gaps were framed as contributing to variability in practice standards, uneven adoption of evidence-based care, and inconsistent engagement in advocacy and professional advancement.

### Variation in Professional Identity Formation

Many respondents observed substantial variability in how audiologists conceptualize their professional role, noting that some clinicians embrace doctoral-level responsibility and autonomy, while others operate within more narrowly defined technical or transaction-focused roles. Participants suggested that this divergence often emerges early in training and is reinforced by clinical placement environments that model inconsistent expectations regarding scope, decision-making, and professional accountability.

### Role of Clinical Training Environments and Preceptors

Respondents frequently identified clinical training sites and preceptors as primary agents of professional socialization. Participants expressed concern that variability in preceptor expectations and site quality leads to inconsistent professional formation among trainees. Several respondents advocated for standardized credentialing of training sites, clearer expectations for preceptors, and stronger alignment between academic curricula and contemporary clinical practice norms. In this context, inadequate professional socialization was viewed as a structural issue rather than an individual failing.

### Need for Postgraduate Training and Structured Career Pathways

There was strong support for the development of formal postgraduate training pathways, including residencies, fellowships, and specialty certification models. Respondents argued that the absence of structured postgraduate progression limits not only advanced clinical mastery but also the reinforcement of professional identity, leadership norms, and accountability to standards of care. Comparisons were frequently drawn to other health professions in which postgraduate training serves as a mechanism for consolidating professional autonomy and responsibility.

### Leadership, Accountability, and Cultural Expectations

Participants highlighted the need for intentional leadership development and clearer expectations regarding professional conduct, ethical practice, and engagement in collective professional goals. Several respondents noted that without explicit socialization around leadership, quality improvement, and advocacy, many audiologists remain disengaged from efforts to advance the profession. This disengagement was described as contributing to fragmentation and slowing progress toward autonomy.

### Intergenerational Dynamics and Sustaining Momentum

Respondents frequently commented on generational differences in expectations and engagement. While students and early-career audiologists were often described as optimistic regarding the future of the profession, participants emphasized that this optimism is shaped by the perspectives, behaviors, and mentorship of experienced practitioners. Professional socialization was, therefore, viewed as extending beyond formal education to include ongoing mentorship, continuing professional development, and reinforcement of shared values across the career span.

### Alignment Statement

Collectively, these findings indicate that professional socialization is widely viewed as a critical yet inconsistently realized component of audiology's development as a clinical doctorate. Respondents emphasized that:

- Formation of a doctoral-level professional identity varies across training environments and early career experiences, contributing to uneven expectations regarding autonomy, accountability, and scope of practice.
- Clinical preceptors and practice settings function as primary agents of professional socialization, yet lack standardized expectations, oversight, and alignment with contemporary professional goals.
- The absence of structured postgraduate training pathways limits both advanced clinical development and reinforcement of professional norms.
- Leadership development, mentorship, and explicit discussion of professional responsibility are underemphasized within current education and practice models.
- Sustained professional socialization requires reinforcement across the career span rather than reliance on predoctoral education alone.

Together, these perspectives suggest that advancing professional socialization within the Audiology 2050 framework will require intentional alignment of education, clinical training environments, mentorship structures, and leadership development to support consistent formation of autonomous, accountable audiologists.

**5. Audiologists are advocates and activists for the profession**

Respondents consistently emphasized advocacy and professional activism as necessary—but unevenly realized—components of audiology's progression toward autonomy. Across responses, participants described broad agreement on the importance of advocacy, coupled with concern that fragmented engagement, organizational mistrust, and inconsistent participation have limited the profession's collective influence. Advocacy was framed not solely as legislative activity, but as a cultural expectation tied to professional identity and responsibility.

### Coordination Across a Fragmented Advocacy Landscape

A dominant theme involved concern that audiology lacks a cohesive and coordinated advocacy presence—a concern respondents framed not as criticism of any individual organization, but as recognition that structural fragmentation across practice settings, professional affiliations, and individual allegiances may dilute the profession's effectiveness in policy and regulatory arenas. Participants described difficulty navigating multiple, well-intentioned but loosely coordinated initiatives, with inconsistent priorities limiting the profession's collective policy traction.

### Advocacy as a Shared Professional Responsibility

Participants emphasized that advocacy should not be confined to organizational leadership or a small subset of highly engaged members but should be understood as a professional obligation inherent to doctoral-level practice. Many respondents suggested that progress toward autonomy depends on the profession treating advocacy as a shared, long-term responsibility rather than a function of individual circumstance—current employer, practice setting, or business model. In this framing, uneven engagement was viewed less as an individual shortcoming than as a structural condition: without mechanisms that distribute advocacy responsibility broadly across the profession, participation will continue to depend on which individuals happen to be positioned and motivated to engage.

### Trust, Transparency, and Governance Concerns

Respondents repeatedly highlighted transparency and trust as preconditions for sustained advocacy. Several participants emphasized the need for clarity regarding how professional input is solicited, represented, and acted upon, noting that ambiguity on these points can erode confidence in collective initiatives. Perceptions that some initiatives receive disproportionate visibility or support relative to others were described as potentially alienating, reinforcing calls for governance structures that are inclusive and seen as fair across the profession.

### From Vision to Action: Implementation and Accountability

Numerous respondents observed that although professional objectives such as Audiology 2050 receive substantial conceptual support, advocacy efforts often are amorphous rather than translating into concrete implementation. Participants called for clearer implementation pathways, defined milestones, and accountability mechanisms to ensure progress toward stated goals. Advocacy, in this context, was framed as an ongoing process requiring sustained engagement, resource commitment, and iterative evaluation rather than episodic campaigns.

### Grassroots Engagement and Professional Momentum

Respondents emphasized the importance of grassroots participation in maintaining advocacy momentum. Students and early-career audiologists were frequently described as entering the profession with strong motivation to contribute; however, participants noted that this enthusiasm can diminish in environments lacking visible progress, inclusive leadership, or meaningful opportunities for engagement. Respondents highlighted the importance of mentorship, communication, and pathways for participation to sustain professional momentum across career stages.

### Alignment Statement

Collectively, these findings indicate that advocacy and professional activism are widely recognized as essential to advancing audiology's autonomy yet are inconsistently realized across the profession. Respondents emphasized that:

- Advocacy is a core professional responsibility associated with doctoral-level practice rather than an optional or organization-specific activity.
- Engagement in advocacy varies across individuals and practice settings, limiting sustained momentum and policy impact.
- Fragmented advocacy efforts and parallel initiatives dilute the profession's effectiveness in regulatory and legislative arenas.
- Trust, transparency, and clarity regarding representation are necessary to sustain broad-based engagement.
- Opportunities for grassroots participation and leadership development are critical to translating professional consensus into coordinated action.

Together, these perspectives suggest that advancing advocacy within the Audiology 2050 framework will require intentional cultivation of shared professional responsibility for policy engagement, supported by coordinated structures that enable collective action while respecting organizational diversity.

**6. Achieve Medicare LLP status**

Because current Medicare statutes within the Social Security Act do not classify audiologists as Limited Licensed Practitioners, respondents consistently identified achieving Medicare LLP status for audiologists as the pivotal structural barrier to professional autonomy. (Audiologists are the only clinical doctoring professionals who are not recognized under Medicare Part B statutes as either physicians, practitioners, or therapists.) Across respondents, the absence of Medicare LLP status was framed as the central constraint affecting scope of practice, reimbursement, workforce utilization, and clinical independence. This essential change to Medicare policy was described not merely as a payment mechanism, but as a defining signal of audiology's status within the broader healthcare system.

### Medicare Classification as a Structural Constraint

A dominant theme centered on the continued classification of audiologists as diagnostic suppliers rather than autonomous practitioners under Medicare. Respondents emphasized that this classification reinforces physician dependency for referrals and billing, limits direct patient access, and constrains audiologists' ability to practice independently—particularly in private practice and community-based settings. Several participants characterized this structure as incompatible with doctoral-level education and misaligned with the recognition afforded to other clinical doctoring professions.

### LLP Status as Foundational to Autonomy and Access

Participants consistently characterized LLP status as foundational rather than incremental. Respondents suggested that without Medicare recognition as autonomous practitioners, efforts to expand the scope of practice, implement evidence-based care, or redesign workforce models would remain structurally constrained. Medicare LLP status was framed as enabling direct access, independent billing, and clearer professional standing within healthcare systems, particularly for older adults and populations disproportionately reliant on Medicare coverage.

### Impact on Workforce Utilization and Practice Models

Respondents linked Medicare policy directly to workforce utilization and sustainability. Several noted that the inability to bill Medicare independently restricts where and how audiologists can practice, discouraging private practice ownership and reinforcing employment in physician-owned or institutionally constrained settings. This dynamic was described as limiting professional autonomy and contributing to dissatisfaction among clinicians seeking greater control over care delivery and practice structure.

### Reimbursement, Value Recognition, and Professional Credibility

Participants emphasized that Medicare LLP status carries symbolic and practical implications beyond payment rates. Respondents described LLP recognition as a signal to commercial payers, healthcare administrators, and policymakers regarding audiology's clinical legitimacy and value. Without LLP status, audiologists reported difficulty demonstrating the value of professional services independent of product sales, reinforcing perceptions of audiology as a technical support rather than a clinical discipline.

### Evaluation and Management Coding as a Structural Prerequisite

Respondents identified a specific and consequential gap in the current reimbursement architecture: the absence of evaluation and management (E&M) coding authority under Medicare. Participants noted that existing reimbursement rates for audiologic diagnostic procedures—when considered in isolation from device-related revenue—are insufficient to sustain independent practice. Under current Medicare fee schedules, reimbursement for comprehensive audiologic evaluation generates revenue inadequate to cover the basic operational costs of a solo practice, before accounting for overhead, staffing, and patient cost-sharing obligations. Respondents argued that E&M office-visit coding, analogous to that available to other LLP-recognized professions, would provide a sustainable service-based revenue foundation that is currently unavailable to audiologists regardless of practice setting. In this framing, achieving Medicare LLP status must be understood as encompassing not only reclassification of audiologists as autonomous practitioners, but also the corresponding expansion of billable service categories that allow professional-level care to be economically viable independent of product transactions.

### Policy Momentum and Collective Responsibility

While many respondents expressed optimism about legislative efforts to achieve Medicare LLP status, they also voiced frustration with the pace of progress. Participants emphasized that translating policy proposals into enacted reform will require sustained advocacy and long-term commitment across the profession. Several respondents viewed the profession's ability to sustain that commitment—more than the legislative outcome itself—as the clearer test of its capacity to act collectively in pursuit of autonomy.

### Alignment Statement

Collectively, these findings indicate that Medicare LLP status is widely viewed as the critical structural requirement for audiology's evolution as an autonomous clinical profession. Respondents emphasized that:

- The current Medicare classification of audiologists as diagnostic suppliers, rather than autonomous practitioners, is misaligned with doctoral-level education and clinical responsibility.
- Absence of LLP status limits direct patient access, independent billing, and full utilization of audiologists' scope of practice, particularly for older adults and Medicare-dependent populations.
- Medicare policy functions as a downstream determinant of professional legitimacy, influencing commercial payer behavior, health system integration, and state-level scope implementation.
- Lack of LLP recognition constrains practice models, reinforcing physician dependency and limiting the viability of independent and community-based audiology services.
- Achievement of LLP status is necessary but not sufficient, with its impact contingent upon concurrent alignment of scope of practice, evidence-based standards, and workforce infrastructure.

Together, these perspectives suggest that achieving Medicare LLP status within the Audiology 2050 framework represents a policy inflection point—one that amplifies upstream alignment with implementation or a profession that remains constrained in its absence.

**7. Primary care entry point for auditory and vestibular conditions**

Respondents addressed the concept of audiologists serving as a primary care entry point for auditory and vestibular conditions with a mix of support, caution, and conditional endorsement. Across responses, participants agreed that audiologists are well positioned to function as the initial point of contact for hearing- and balance-related concerns. However, this role was consistently framed as contingent upon prior achievement of scope alignment, standardized practice, workforce infrastructure, effective advocacy, and Medicare LLP reclassification.

### Conceptual Support Anchored in Domain-Specific Expertise

A substantial number of respondents supported the concept of audiologists serving as primary entry points, provided responsibilities were clearly delineated within the domains of auditory and vestibular care. Participants emphasized that audiologists possess specialized training in differential diagnosis, longitudinal management, and rehabilitative decision-making for hearing and balance disorders. In this framing, primary care entry point status was viewed as an extension of existing competencies rather than an expansion into generalized medical care.

### Concerns Regarding Overreach and Role Clarity

At the same time, respondents frequently cautioned against language that could be interpreted as positioning audiologists as providers of whole-body primary care. Multiple participants noted that such framing could elicit resistance from other healthcare professions and potentially undermine audiology's credibility. Respondents emphasized the importance of precise terminology, underscoring that entry point status should be limited to auditory and vestibular complaints and aligned with licensure, training, and established referral pathways.

### Dependence on Structural Preconditions

Participants consistently described primary care entry point status as dependent on the resolution of upstream structural barriers. Respondents noted that without a modernized scope of practice, standardized evidence-based care, a tiered workforce, and Medicare LLP status, audiologists would lack the authority and infrastructure necessary to function effectively as entry points. In this context, entry point status was portrayed not as a standalone initiative, but as a downstream outcome of broader system alignment.

### Reimbursement and Practice Model Constraints

Respondents highlighted reimbursement as a critical determinant of feasibility. Participants observed that achieving reimbursement levels for professional services sufficient to support independent practice—particularly in the absence of reliance on hearing aid sales—is essential before entry point status can be realized across many settings. Entry point roles were viewed as incompatible with models in which audiologists remain financially dependent on physician-owned practices or product-based revenue streams.

### Public Understanding and System Integration

Finally, respondents emphasized that successful implementation of an entry point model would require improved public understanding of audiologists' roles and clearer integration within healthcare systems. Participants noted that confusion among patients, payers, and other providers regarding distinctions between audiologists, otolaryngologists, hearing aid specialists, and assistants limits acceptance of audiologists as first-contact providers. Addressing this gap was viewed as essential for realizing the entry point vision.

### Alignment Statement

Collectively, these findings indicate that audiologists serving as a primary care entry point for auditory and vestibular conditions are broadly supported in principle but consistently framed as a downstream outcome contingent on prior structural alignment across the Audiology 2050 framework. Respondents emphasized that:

- Audiologists possess specialized training in the evaluation, differential diagnosis, and longitudinal management of hearing and balance disorders, positioning them to function as first-contact providers for auditory and vestibular complaints.
- Support for entry point status is limited to auditory and vestibular conditions, with caution against language or models that could be interpreted as whole-body primary care or professional overreach.
- Realization of an entry point role depends on the modernization of the scope of practice, standardized evidence-based care, and Medicare LLP status to ensure appropriate authority, reimbursement, and system recognition.
- Current reimbursement structures, physician-dependent practice models, and product-based revenue incentives are incompatible with sustainable entry point care.
- Public, payer, and interprofessional understanding of audiologists' roles remains insufficient, limiting acceptance of audiologists as first-contact providers within healthcare systems.

Together, these perspectives suggest that positioning audiologists as a primary care entry point for auditory and vestibular conditions represents the culminating outcome of the Audiology 2050 framework—achievable only through coordinated progress across scope, standards, workforce design, professional socialization, advocacy, and federal recognition.

## Discussion

The findings presented in this study reveal a profession that is not lacking vision, motivation, or technical expertise, but constrained by persistent structural misalignment. Consistent with the structural account developed in companion analyses of audiology wages, taxonomy, and professional status, the qualitative data confirm that the profession's most frequently cited challenges—scope limitations, inconsistent practice standards, education and debt, workforce strain, reimbursement barriers, and constrained autonomy—are not independent problems. Rather, they represent interdependent system components that must mature in a coordinated sequence to achieve sustainable professional autonomy. What the qualitative evidence adds is the practitioner-level texture of that misalignment: the lived experience of dependency relationships that the structural analyses document in economic terms.

### Structural Dependency Across Audiology 2050 Domains

A central insight emerging from this analysis is the presence of clear dependency relationships among the seven Audiology 2050 domains. Across domains, scope of practice emerges as the foundational enabling condition upon which other objectives depend. Without scope alignment consistent with doctoral-level training, efforts to standardize evidence-based practice, redesign workforce models, or achieve Medicare LLP status remain structurally constrained. This relationship was not asserted abstractly; it was embedded in respondents' descriptions of professional experience across settings.

Market-level evidence corroborates this structural dependency. 2 When reimbursement constraints make participation in third-party administrator (TPA) channels economically untenable for audiology practices, the rational individual response—strategic TPA divestment—produces an aggregate outcome that is collectively harmful: technically trained hearing aid specialists expand into the referral infrastructure that audiology vacates, reshaping patient entry pathways in ways that are difficult to reverse. This dynamic illustrates why scope and reimbursement reform are not merely economic objectives; they are preconditions for maintaining audiology's position within the care delivery infrastructure that routes patients to providers in the first place.

The structural dependency analysis points toward a more fundamental cycle underlying these market dynamics—one that expert respondents described, in various forms, across multiple Audiology 2050 domains. Because audiologists cannot independently bill for their cognitive clinical labor at the doctoral level, practice revenue becomes structurally dependent on product transactions to remain economically viable; because revenue is anchored to device sales rather than independently priced clinical services, the profession is perceived by payers, policymakers, and the public as operating in a retail rather than clinical capacity; and because that perception persists, the billing reclassification that would establish a nonsales-based revenue foundation remains elusive. Each element reinforces the others. This cycle is not incidental to audiology's classification challenges—it is the mechanism through which those challenges reproduce themselves across legislative sessions, reimbursement cycles, and professional generations. Interrupting this cycle requires not a single reform but the coordinated structural alignment of this issue documents: taxonomy modernization that enables E&M billing, reimbursement recognition that establishes clinical revenue independent of device transactions, and workforce models that demonstrate doctoral-level productivity without product dependence.

These dependency relationships are summarized in Fig. 2 , which maps the interconnectedness of the seven Audiology 2050 domains discussed in this analysis. Scope of practice is positioned as the sole foundational domain, consistent with respondents' characterization of scope as the enabling condition upon which standardized evidence-based practice and workforce design directly depend (solid lines). Professional socialization and advocacy are linked by a reciprocal, indirect relationship reflecting their function as cultural infrastructure rather than as formal regulatory prerequisites (dotted line); both nonetheless feed forward into the achievement of Medicare LLP status, which the analysis identifies as the critical structural inflection point for audiology's professional advancement. Primary care entry point status is depicted as the culminating, profession-wide outcome, with dotted lines tracing the indirect but consequential influence of scope of practice and evidence-based practice on this endpoint across the full dependency chain. Fig. 2 is intended as a structural reference: it does not imply that distal domains are unrelated, but that their influence on one another is mediated through the intervening domains depicted.

**Fig. 2:**
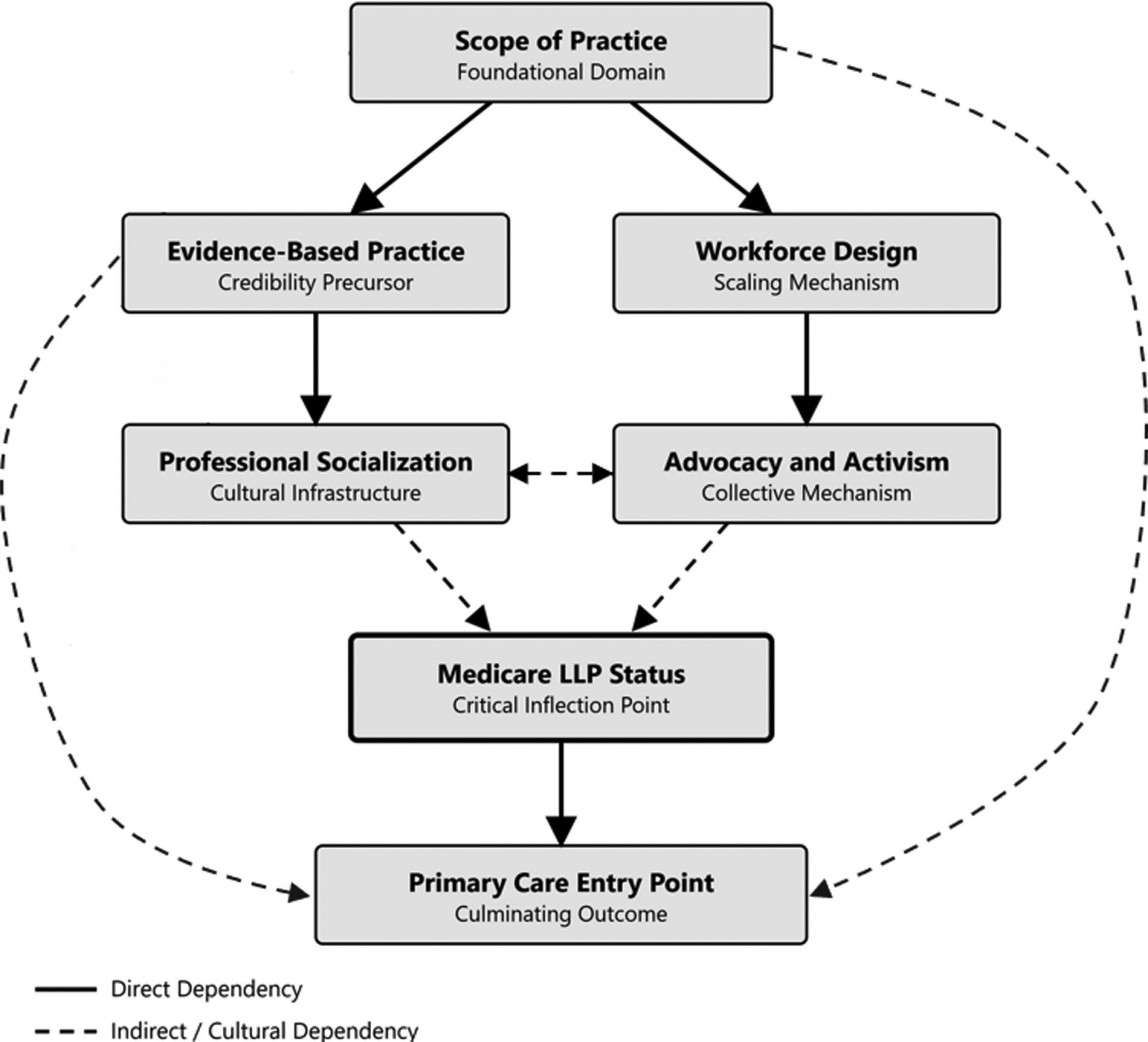
Interconnectedness of the seven Audiology 2050 domains. Solid lines indicate direct structural dependencies; dotted lines indicate indirect or cultural dependencies.

Similarly, standardized, evidence-based practice emerged as a necessary precursor to claims of autonomy and expanded authority. Respondents recognized that professional credibility—particularly in the eyes of payers, policymakers, and interprofessional partners—depends on demonstrable consistency in care delivery. In this framing, variability in practice quality undermines not only patient outcomes but also the profession's negotiating position in efforts to modernize scope and reimbursement.

### Workforce Design as a Scaling Mechanism, not a Substitute

The analysis further highlights that workforce sustainability is best understood as a utilization problem rather than a supply problem. Respondents did not describe a simple shortage of audiologists; instead, they emphasized role compression, underutilization of doctoral expertise, and burnout resulting from inefficient care models. Within this context, service extenders were consistently framed as a scaling mechanism that enables, rather than replaces, autonomous practice.

Importantly, respondents linked workforce redesign to upstream domains. Without standardized practice expectations, clearly defined scopes, and reimbursement structures that support professional services, extender models risk amplifying variability in care rather than alleviating workforce strain. Thus, workforce expansion was viewed as dependent on prior alignment across scope, standards, and payment.

### Professional Socialization and Advocacy as Cultural Infrastructure

Beyond formal structures, respondents emphasized the importance of professional culture in sustaining reform. Gaps in professional socialization—particularly during clinical training and early career transitions—were described as contributing to uneven adoption of evidence-based practice and inconsistent engagement in advocacy. These findings suggest that autonomy cannot be achieved solely through policy change; it also requires intentional cultivation of professional identity, leadership norms, and shared expectations.

Advocacy and activism were similarly framed as dependent behaviors rather than isolated activities. Respondents indicated that sustained advocacy emerges most readily when audiologists view autonomy as a shared, profession-wide responsibility rather than a matter of individual circumstance. Structural fragmentation across practice settings and professional affiliations was repeatedly identified as a limiting factor, reinforcing the need for coordinated governance and shared strategic direction.

### Medicare LLP Status as a Structural Inflection Point

Among all seven domains, Medicare LLP status emerged as the critical inflection point for change—both symbolically and practically. Respondents consistently described LLP classification as a determinant of professional legitimacy, influencing how audiologists are perceived by commercial payers, healthcare systems, and policymakers. However, the analysis also clarifies that LLP status alone is insufficient to produce autonomy. Without concurrent modernization of scope, standardization of practice, and workforce alignment, Medicare reclassification risks underdelivering on its promise. In this sense, LLP status functions less as a destination than as a multiplier—amplifying the effects of upstream alignment while remaining constrained by its absence.

### Primary Care Entry Point as a Downstream Outcome

Finally, the role of audiologists as a primary care entry point for auditory and vestibular conditions was consistently framed as an outcome rather than a starting premise. Respondents broadly supported this role in principle, but only under conditions of clear role definition, adequate authority, standardized care delivery, and sustainable reimbursement. This position reinforces the notion that entry point status is contingent on successful execution of preceding domains, rather than an independent policy objective.

### Audiology 2050 as a Profession-Wide, Implementation-Oriented Roadmap

Taken together, these findings support a roadmap to achieve the Audiology 2050 goals. Rather than functioning primarily as an organizational initiative, Audiology 2050 operates most effectively as a sequenced, implementation-oriented strategic roadmap for structural alignment. The dependencies articulated by respondents suggest that selective or piecemeal implementation is unlikely to yield durable progress. Instead, advancement toward autonomy requires coordinated movement across domains, with early emphasis on foundational elements that enable downstream reform.

Although Audiology 2050 was first articulated by the ADA, the themes identified in this analysis reflect profession-wide concerns that transcend organizational boundaries. This is evidenced not only by the breadth of commentary received, but by its distribution: respondents drawn from all eight occupational segments depicted in Fig. 1 offered substantive commentary on every one of the seven Audiology 2050 domains, despite the survey's open-ended format placing no expectation on participants to address each domain individually. With 79% (41/52) of invited subject matter experts responding, and every occupational segment represented in commentary across every domain, the convergence observed here is unlikely to reflect the perspective of any single professional vantage point. Rather, it suggests that the framework's seven priorities are recognized as consequential across the full structural diversity of the profession. As such, the roadmap developed in this manuscript should be understood as shared infrastructure capable of guiding collective action across education, practice, policy, and advocacy.

Notably, some respondents with backgrounds in strategic planning and continuous improvement observed that translating the Audiology 2050 framework into durable outcomes will require more than the identification of strategic domains. Effective implementation will depend on establishing a current-state baseline, conducting systematic root-cause analysis of the structural gaps between present conditions and desired outcomes, and defining process-level metrics that enable iterative evaluation and course correction. In the absence of short-cycle accountability mechanisms—alongside the long-term strategic priorities already articulated—the framework risks functioning as a current statement of aspirational goals rather than a driver of measurable change. Operationalizing the roadmap with discipline commensurate with improvement of science standards represents a necessary next step for the profession.

### Implications for Strategic Prioritization

The findings suggest that prioritization in audiology should be understood not as ranking initiatives by importance, but as sequencing interventions by dependency. Scope modernization, practice standardization, and workforce redesign function as enabling conditions for reimbursement reform, Medicare LLP reclassification, and expanded care models. Efforts that bypass these dependencies risk reinforcing the very constraints they seek to overcome.

In this light, this article offers not only a vision of the future but a framework for disciplined execution—one that aligns professional aspirations with the structural changes required to realize them ( Fig. 3 ). If the dependencies identified above remain unaddressed, the profession's trajectory can be projected with reasonable clarity.

**Fig. 3:**
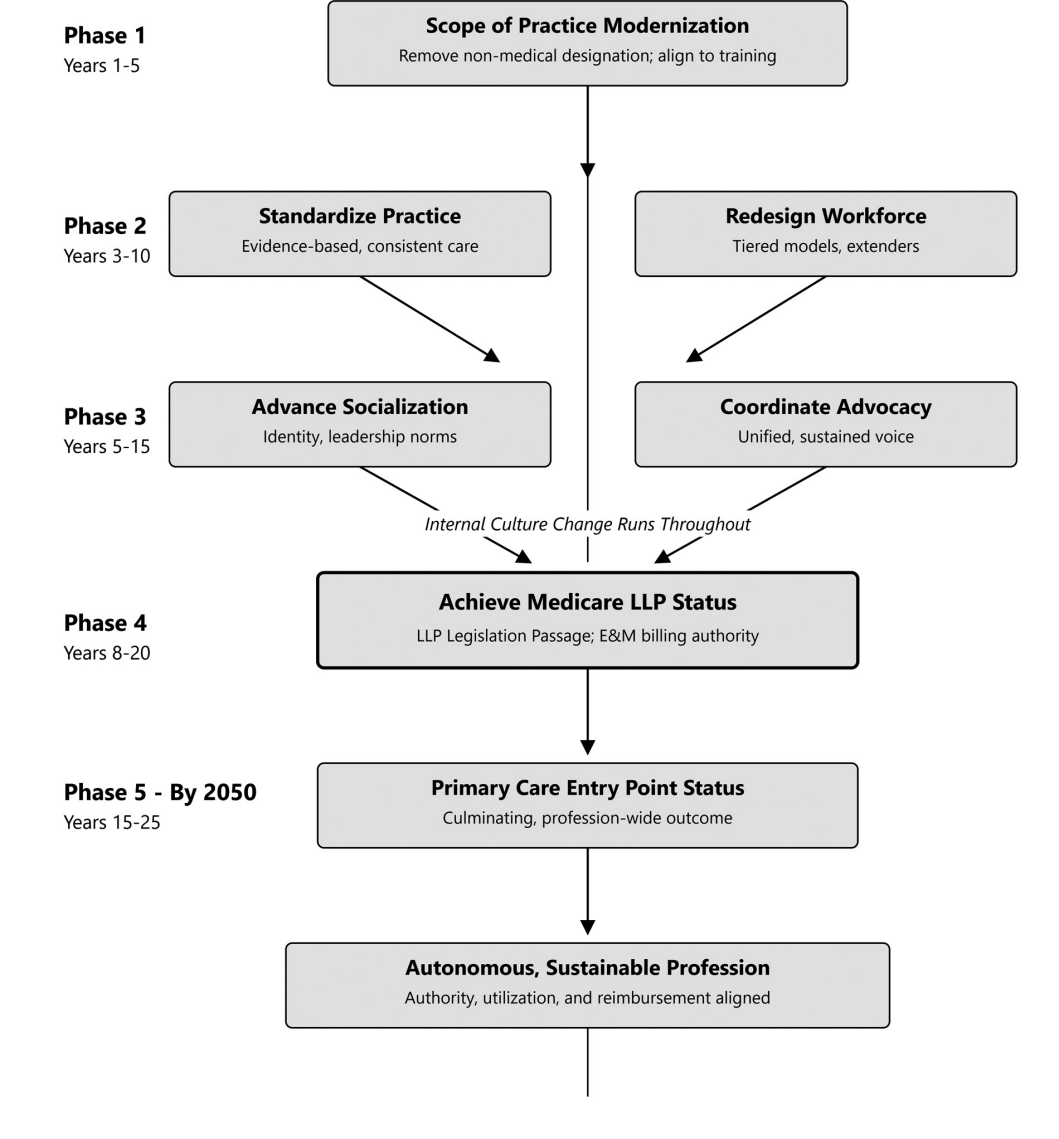
Proposed Audiology roadmap to 2050. Phases reflect general time sequencing based on upstream and downstream dependencies among domains; overlapping year ranges indicate intentionally concurrent, coordinated reform rather than strict sequential completion.

### The Cost of Inaction: A Plausible 2050 Trajectory

Inaction is often framed as neutrality. In the context of healthcare workforce policy, however, continuation of existing economic, educational, and regulatory structures is not a neutral choice—it is a structural condition with predictable consequences. The analyses presented throughout this special issue are consistent with those structures being increasingly misaligned with audiology's training requirements, scope of practice, and societal value. If current conditions persist without structural intervention, the evidence supports a foreseeable trajectory for the profession by mid-century.

Under status quo assumptions, audiology in 2050 is likely to occupy a progressively narrower role within hearing healthcare delivery. Despite rising population-level demand driven by both population growth and aging demographics, combined with the expanding recognition of hearing loss as a modifiable risk factor for cognitive decline and broader health outcomes, the profession's capacity to meet that demand is unlikely to scale proportionately. Workforce growth has lagged behind comparable health professions, and there is little evidence that current educational and reimbursement models will reverse this trend absent structural reform.

Economic signals further reinforce this projection. Longitudinal wage and earnings data indicate that the transition to doctoral-level entry has not produced sustained divergence in compensation relative to peer professions with lower terminal degree requirements. When coupled with persistently high educational debt, this misalignment places downward pressure on labor supply, limits geographic mobility, and constrains the profession's ability to attract and retain talent. Over time, such pressures risk transforming audiology into a profession characterized by high training intensity but diminishing economic leverage.

As workforce capacity tightens, service delivery models are likely to adapt in ways that further marginalize audiology's role in system-level decision-making. Tasks may increasingly be unbundled, delegated, or automated, with audiologists positioned as downstream service providers rather than upstream clinical leaders. In this scenario, influence over care pathways, reimbursement structures, and innovation design shifts toward organizations and stakeholders better aligned with prevailing economic incentives—not necessarily those with the deepest expertise in hearing and balance care.

Current private practice market data corroborate this trajectory. As documented in companion analysis of audiology's professional status within this issue (Article 7, Health Economics), new patient acquisition in private practice hearing care declined in 2025 for the first time on record despite continued demographic expansion of the population aged 65 and older, with audiology practices ceding referral infrastructure to hearing aid specialist providers as a rational response to reimbursement structures that make TPA participation economically untenable. 2 This is not a projected outcome—it is a present-state condition, and one that illustrates precisely how inaction compounds structural misalignment into market-level displacement.

This trajectory does not imply a decline in clinical competence or professional commitment among audiologists. It is consistent with a structural decoupling between the profession's educational investment and the healthcare system's valuation of its services—a pattern the economic evidence reviewed in this issue supports across multiple dimensions. If the structural conditions documented here remain unchanged, the evidence predicts progressive narrowing of audiology's institutional authority within hearing healthcare delivery, independent of the clinical quality audiologists bring to that role.

This outcome is not structurally determined. It is the projected consequence of current policy and market conditions under status quo assumptions—and those assumptions are modifiable. The analytical value of projecting this trajectory is not motivational but diagnostic: it identifies which structural variables, if changed, would alter the projection, and in what sequence.

An additional and underappreciated contributor to this trajectory warrants explicit acknowledgment: inertia internal to the profession itself. Respondents described a segment of the audiology workforce that actively operates below its full doctoral scope—not due to structural constraints, but by choice—and that may resist changes perceived as destabilizing to established practice models. This dynamic is distinct from the external policy and reimbursement barriers that have received greater attention in the literature. Structural reform, however necessary, will remain insufficient if a meaningful portion of the profession opts out of the doctoral-level identity and responsibility that full autonomy requires. Realizing the Audiology 2050 goals will therefore demand not only external policy change but intentional internal culture change—one that makes practicing to the full scope the professional expectation rather than the exception.

## Conclusion and Implications

The findings presented in this study underscore a central implication confronting audiology: the profession's future will be determined not by isolated reforms, but by coordinated structural alignment across education, workforce design, legislation, regulation, and federal policy. The analyses synthesized here suggest that Audiology 2050 is not simply a vision of what the profession could become but supports a roadmap for avoiding a future in which audiology's clinical expertise persists without corresponding authority, utilization, or influence. In fact, the analyses indicate that expert perspectives converge on a clear message—professional autonomy is achievable, but only if dependencies among foundational domains are acknowledged and addressed in sequence.

### Implications for Federal Policy: Medicare LLP Legislation as a Structural Enabler

At the federal level, today's Medicare Audiology Access Improvement Act of 2025 (MAAIA), which includes Medicare reclassification of audiology as an LLP profession, represents a critical opportunity to recalibrate audiology's role within the healthcare system. Respondents consistently framed Medicare LLP status as both a practical and symbolic inflection point, with implications extending beyond reimbursement to professional legitimacy and system integration. Importantly, the findings suggest that LLP legislation should be understood as an enabling statute rather than a standalone solution. Its impact will be maximized only if accompanied by a modernized scope of practice and demonstrable adherence to standardized, evidence-based care. In this sense, LLP recognition functions as a multiplier—amplifying upstream alignment while remaining constrained by its absence. The structural dependency analysis developed here suggests that federal reclassification is most likely to produce durable economic effects when accompanied by upstream scope and standards alignment—not as a standalone intervention.

### Implications for State Modernization: Scope as Foundational Infrastructure

State-level modernization of the scope of practice emerges as a prerequisite for nearly every downstream objective articulated by respondents. Variability in scope across jurisdictions and practice settings continues to limit workforce utilization, impede standardized care, and complicate federal policy implementation. The findings suggest that scope reform should prioritize alignment with doctoral-level competencies, clarity of clinical authority, and consistency across states. Such alignment would not only support federal initiatives like Medicare LLP status but also create the regulatory conditions necessary for tiered workforce models, evidence-based reimbursement, and primary care entry point roles. The dependency structure identified in this analysis predicts that federal recognition without state scope alignment will produce uneven implementation—a testable proposition that future workforce data should be able to evaluate.

### Implications for AuD Curriculum Reform: Preparing for the Profession That Is Needed

The transition to the AuD degree represented a pivotal moment in audiology's evolution, yet the findings indicate that educational reform has not been fully matched by corresponding changes in authority, utilization, or professional socialization. Respondents emphasized that future curriculum reform must move beyond technical competency to include preparation for autonomous clinical decision-making, leadership, advocacy, and system-level practice. This includes stronger integration of evidence-based care implementation, exposure to tiered workforce models, and explicit professional socialization aligned with autonomy and accountability. Curriculum reform, in this framing, functions as a structural input to the dependency sequence—one whose effects on workforce readiness and professional socialization are observable and evaluable over time.

### Professional Alignment as a Necessary Condition for Progress

An additional implication of these findings is the necessity for greater alignment across professional organizations. Because the structural challenges facing audiology—scope of practice, workforce utilization, reimbursement, and federal recognition—are interdependent, progress in any single domain is unlikely to be sustained in the absence of cross-organizational coordination. While professional organizations may differ in tactics, emphasis, and governance, the data suggest that alignment around shared goals, sequencing, and implementation is a necessary condition for realizing the Audiology 2050 goals and achieving durable professional autonomy.

### Coordinated Implementation as the Path Forward

Collectively, these implications are consistent with a systems-level interpretation of the evidence: progress in any single domain is unlikely to produce durable change unless the dependency relationships identified in this analysis are respected in sequencing. The structural conditions examined across this special issue—reimbursement authority, scope alignment, workforce design, and educational preparation—are interdependent inputs, not independent levers. Interventions that treat them as separable are predicted by this framework to produce limited and potentially temporary effects.

Although Audiology 2050 was first articulated by the ADA, the qualitative evidence synthesized here indicates that its structural logic resonates across practice settings, career stages, and organizational affiliations. As an analytic framework, it provides a basis for evaluating reform sequencing and measuring whether observable economic signals—earnings trajectories, workforce stability, service valuation—respond in ways consistent with doctoral-level professional standing.

Translating this dependency structure into an actionable timeline, Fig. 3 presents a proposed sequencing across the seven Audiology 2050 domains, organized into four overlapping phases rather than a strict, nonoverlapping waterfall. Phase 1 (years: 1–5) initiates scope-of-practice modernization, the foundational domain on which the remaining phases depend. Phase 2 (years: 3–10) advances standardized, evidence-based practice and workforce redesign in parallel, consistent with the analysis's characterization of these two domains as co-dependent rather than sequential outputs of scope reform. Phase 3 (years: 5–15) advances professional socialization and coordinates advocacy efforts; deliberate overlap with Phase 2 reflects respondents' emphasis that cultural and structural reform must proceed together rather than await one another. Phase 4 (years: 8–20) targets the implementation of LLP legislative status and the establishment of E&M billing authority, consistent with this domain's identification as the critical structural inflection point, thereby solidifying advancement toward an autonomous profession. By 2050 (years: 15–25), sustained progress across all preceding domains is proposed to culminate in audiologists' recognition as a primary care entry point for auditory and vestibular conditions, yielding an autonomous, sustainable profession in which clinical authority, workforce utilization, and reimbursement are structurally aligned. The overlapping phase boundaries are intentional: they depict a coordinated, concurrent reform effort rather than a sequence in which any one domain must be fully resolved before the next is engaged. Internal culture change—the intentional cultivation of professional expectations around full-scope, doctoral-level practice discussed above—is not bound to a single phase but is proposed to run throughout the full roadmap, as this dimension of reform operates continuously alongside, rather than prior to, the structural milestones depicted.

Finally, respondents identified technological change—and AI in particular—as an environmental variable that the Audiology 2050 framework has not yet systematically addressed but must engage as the profession operationalizes toward mid-century. AI-assisted hearing screening, automated audiogram interpretation, diagnostic triage tools, and consumer-facing hearing health monitoring platforms are already entering both clinical and direct-to-consumer markets. Their maturation will reshape demand for audiologic services in ways that are structurally consequential, not merely operationally disruptive.

The dependency structure identified in this analysis suggests that AI's effect on audiology's professional position is not predetermined—it is conditional on whether structural alignment is achieved before AI-driven substitution accelerates. Where scope remains narrowly associated with device dispensing and routine audiometry, the substitutability dynamic documented in this issue predicts that AI-assisted alternatives will absorb precisely those functions. Where audiologists function as diagnosticians of complex auditory and vestibular pathology and clinical interpreters of AI-generated data, the technology becomes a productivity lever rather than a competitive threat. The Audiology 2050 framework, if operationalized with disciplined attention to sequencing, provides the structural conditions under which the latter outcome is more probable than the former—a prediction that the profession's trajectory over the next decade should be able to test.
